# Risks and complications of transurethral resection of bladder tumors in patients receiving antiplatelet and/or anticoagulant therapy: a retrospective cohort study

**DOI:** 10.1186/s12894-017-0309-0

**Published:** 2017-12-12

**Authors:** Tsuzumi Konishi, Satoshi Washino, Yuhki Nakamura, Masashi Ohshima, Kimitoshi Saito, Yoshiaki Arai, Tomoaki Miyagawa

**Affiliations:** 1 Department of Urology, Jichi Medical University Saitama Medical Center, 1-847 Amanuma-cho, Omiya-ku, Saitama, 330-8503 Japan; 2 Department of Urology, Nishi-Omiya Hospital, 1-1173 Mihashi, Omiya-ku, Saitama, 330-0856 Japan

**Keywords:** Anticoagulant, Antiplatelet, Turbt

## Abstract

**Background:**

Information on the safety of transurethral resection of bladder tumors (TURBT) in patients receiving anti-thromboembolic drugs is currently lacking. This study aimed to evaluate the clinical safety of TURBT in patients receiving anti-thromboembolic agents compared with patients not taking these agents and patients who interrupted their use perioperatively.

**Methods:**

We retrospectively analyzed data for patients who underwent TURBT at Jichi Medical University Saitama Medical Center from September 2013 to August 2016.Patients who underwent surgery while receiving antiplatelet and/or anticoagulant drugs were allocated to the continuation group, those who interrupted these drugs comprised the interruption group, and those who did not use these agents were designated as the control group. We compared the patient characteristics, hemoglobin levels, and complications among the three groups.

**Results:**

A total of 174 patients were analyzed including 19, 18, and 137 in the continuation, interruption, and control groups, respectively. There were no significant differences in patient and tumor characteristics, apart from age, among the three groups. Decreases in hemoglobin levels were similar in the continuation, interruption, and control groups (−0.50 g/dl, −0.40 g/dl, and −0.50 g/dl, respectively).Significantly more patients in the continuation group experienced clot retention compared with the control group (21% vs 5%, *p* = 0.03). Large tumor size tended to be a risk factor for clot retention in the continuation group (*p* = 0.07). No patient in the continuation or interruption group required blood transfusion, compared with two patients (1%) in the control group. No patients in any of the groups experienced cardiovascular events during their hospital stay or required rehospitalization for hematuria after discharge.

**Conclusions:**

TURBT can be performed safely in patients who continue to take antiplatelet and/or anticoagulant agents, without increasing the risks of severe hemorrhage and blood transfusion. However, the risk of postoperative clot retention may be increased in these patients.

## Background

Urologists are encountering increasing numbers of patients with multiple comorbidities associated with the progressive aging of the population. These include coronary artery disease requiring percutaneous coronary arterial intervention with angioplasty, together with the placement of bare metal (BMS) or drug-eluting stents (DES), and cardiac dysrhythmias such as valvular heart disease, deep vein thrombosis, or atrial fibrillation [1]. International guidelines recommend dual antiplatelet (AP) therapy (DAPT) for ≥4 weeks after implantation of BMS, and for 6–12 months after implantation of DES [2, 3], while anticoagulant (AC) agents are recommended in patients with cardiac dysrhythmias. Withdrawal of AP and/or AC agents is associated with a significantly increased risk of cardiac ischemic and/or thromboembolic events [4]. Surgeons, physicians, and patients thus face the dilemma of stopping these agents perioperatively to reduce the bleeding risk, and continuing them to avoid the risk of cardiovascular and cerebrovascular events [5]. There is currently no consensus among urologists regarding the perioperative management of patients taking AP/AC agents, and information on this issue is lacking [6]. A few recent reports have assessed the safety of continuing to use AP agents during transurethral resection of bladder tumors (TURBT) [5, 7], but there is little information on the safety of TURBT in patients receiving AC or combined AP/AC therapy. This study aimed to evaluate the clinical safety of TURBT in patients receiving AP/AC agents, compared with patients not taking these agents or patients who interrupted their use perioperatively.

## Methods

### Patients

This retrospective observational study was approved by our local institutional review board. Patients who underwent TURBT at Jichi Medical University Saitama Medical Center from September 2013 to August 2016 and who were followed-up for at least 3 months were eligible. Patients who underwent additional procedures at the same time as TURBT or who underwent second TURBT were excluded. Eligible patients were categorized into two groups: patients taking AP and/or AC drugs before surgery (AP/AC group) and those not taking these drugs (control group). Patients in AP/AC group were further categorized into two groups: patients who took AP/AC drugs during surgery (continuation group) and those who interrupted the drugs for the appropriate periods before, during, and after surgery (interruption group). The decision on whether to continue or interrupt AP/AC drugs was made after detailed discussions between patients and doctors (urologists, anesthesiologists, neurologist, and/or, cardiologists), mainly in light of the risk of cardiovascular events.

### Device

TURBT was performed using a UES-40S (Olympus R, Tokyo, Japan) or ESG-400 (Olympus) endoscope.

### Management

In principle, patients were not allowed to walk on the day of surgery, and started to walk the next morning. Blood tests, including hemoglobin levels, were performed on the first postoperative day. Patients were discharged from the hospital after removal of the urethral catheter and spontaneous voiding had been experienced.

#### Study endpoints

Patients’ characteristics, decreases in hemoglobin levels, median catheter-indwelling duration after TURBT, and complications were compared among the three groups. The endpoints of our study were decrease in hemoglobin level and hemorrhagic complications after TURBT. Decreases in hemoglobin levels were determined as [preoperative hemoglobin level – hemoglobin level on first postoperative day]. Preoperative examinations were performed within 2 months before surgery.

### Statistical analysis

Statistical analysis was performed using GraphPad Prism software version 6.0. Data were compared using Student’s *t*-tests, Mann–Whitney U-tests, or χ^2^ tests. All data are shown as medians and ranges. Statistical significance was set at *p* < 0.05.

## Results

### Patients and tumor characteristics

A total of 229 patients were eligible, of whom 31 patients who underwent additional procedures at the same time as TURBT and 24 who underwent second TURBT were excluded. A total of 174 patients were therefore analyzed, including 37 patients in the AP/AC group and 137 in the control group. Among the 37 AP/AC patients, 19 were in the AP/AC continuation group and 18 were in the interruption group.

Patient and tumor characteristics are shown in Table 1. The age in the interruption group was significantly higher than in the control group (*p* = 0.003), but gender, tumor size, tumor number, T classification at TURBT, and de novo/recurrence did not differ significantly among the three groups.

**Table 1:** Patient and tumor characteristics

	AP/AC								
	Continuation (A) *n* = 19, n (%)		Interruption (B) *n* = 18, *n* (%)		Control (C) *n* = 137, *n* (%)		*p* value		
							A vs B	A vs C	B vs C
Median age (range)	77	(57–89)	81	(58–90)	72	(38–90)	0.14	0.12	0.00322
Sex									
Male	17	(88%)	14	(78%)	97	(71%)	0.40	0.10	0.78
Female	2	(12%)	4	(22%)	40	(29%)			
Tumor size									
< 1 cm	10	(53%)	8	(44%)	71	(52%)	0.75	1.0	0.62
≥ 1 cm	9	(47%)	10	(56%)	66	(48%)			
Tumor number									
Single	11	(58%)	12	(67%)	71	(52%)	0.74	0.81	0.32
Multiple	8	(42%)	6	(33%)	66	(48%)			
T classification at TURBT									
pT0–a	8	(42%)	7	(39%)	53	(39%)	0.43	0.71	0.35
pT1	9	(47%)	10	(56%)	59	(43%)			
Min. pT2	2	(11%)	1	(5%)	25	(18%)			
De novo	10	(53%)	10	(56%)	83	(61%)	>0.95	0.62	0.80
Recurrence	9	(47%)	8	(44%)	54	(39%)			

*AP* antiplatelet agents, *AC* anticoagulant agents, *Min* minimum

### Details of medications and reasons for taking AP/AC agents

Among the 19 patients in the continuation group, seven, three, four, and five were taking a single AP, single AC, DAPT, and AP plus AC, respectively, prior to surgery (Table 2). Of the four patients taking DAPT, three interrupted clopidogrel during the perioperative period, and of the five patients taking AP plus AC, one interrupted the AC agent during the perioperative period. None of the other patients interrupted any AP/AC agents. At the time of surgery, 11, three, one, and four patients were taking a single AP, single AC, DAPT, and AP plus AC, respectively.

**Table 2:** Details of medication before surgery

		Continuation, *n* = 19 *n* (%)				Interruption, *n* = 18 *n* (%)		
		Before surgery		During surgery		Before surgery		During surgery
Single agent	Aspirin	7	(37)	11	(58)	11	(61)	–
	Clopidogrel	–		–		1	(5)	–
	AC	3	(16)	3	(16)	3	(17)	–
Combination	DAPT	4	(21)	1	(5)	0	(0)	–
	AP + AC	5	(26)	4	(21)	3	(17)	–

*AP* antiplatelet agents, *AC* anticoagulant agents, *DAPT* dual antiplatelet therapy

A heparin bridge was performed in nine patients in the interruption group, including four, two, and three patients taking a single AP, single AC, and AP plus AC, respectively, prior to surgery. In the heparin-bridged patients, heparin was started at a dose of 15,000 U/day, adjusted to achieve an activated partial thromboplastin time 1.5–2 times the control value, and then discontinued 4 h before surgery. Oral AP and/or AC drugs were re-started after no or little hematuria was achieved postoperatively.

The reasons for taking APs and/or ACs are shown in Table 3. Ischemic heart disease (51%), atrial fibrillation (30%), and cerebral infarction (16%) were the three main reasons.

**Table 3:** Reasons for medication

	Continuation *n* = 19, n (%)		Interruption *n* = 18, *n* (%)		Total *n* = 37, *n* (%)	
IHD	12	(63%)	7	(39%)	19	(51)
AF	5	(26%)	6	(33%)	11	(30)
CI	0	(0%)	6	(33%)	6	(16)
Primary prevention	2	(11%)	1	(6%)	3	(8)
Others	3	(16%)	0	(0%)	3	(8)

*IHD* ischemic heart disease, *AF* atrial fibrillation, *CI* cerebral infarction

### Anesthesia

Among the 19 patients in the continuation group, 17 underwent TURBT under general anesthesia and two under spinal anesthesia. In the interruption group, 11 and seven patients underwent TURBT under spinal and general anesthesia, respectively, while 104 and 33 patients in the control group underwent TURBT under spinal and general anesthesia, respectively.

### Complications

Complications during and after TURBT in the three groups are shown in Table 4. Decreases in hemoglobin levels were similar in all three groups (−0.50 g/dl, −0.40 g/dl, and −0.50 g/dl in the continuation, interruption, and control groups, respectively). Significantly more patients (21%, 4/19) in the continuation group experienced clot retention compared with the control group (5.0%, 7/140) (*p* = 0.03). All four patients with clot retention in the continuation group were only taking aspirin (Table 5). Two of them experienced clot retention during catheterization (postoperative day [POD] 1 and POD3) and required surgical reintervention, while the other two experienced clot retention after catheter removal (POD3 and POD21), which was improved by bladder drainage and catheter replacement. Two (11%) and three patients (2%) in the continuation and control groups required surgical reintervention to stop bleeding, respectively. The median duration of catheter-indwelling was significantly longer in the continuation group (2 days) compared with the control group (1 day) (*p* = 0.03). However, there was no significant difference in hospitalization days after surgery among the three groups. No patient in the continuation or interruption group required blood transfusion, compared with two patients (1%) in the control group. No patients in any of the groups experienced cardiovascular events during their hospital stay, or required rehospitalization for hematuria during the 3 months after discharge.

**Table 4:** Surgical outcomes and complications

	AP/AC								
	Continuation (A) *n* = 19, *n* (%)		Interruption (B) *n* = 18, *n* (%)		Control (C) *n* = 137, *n* (%)			*p* value	
							A vs B	A vs C	B vs C
Median Hb decrease g/dL (range)	−0.5	(−−2.8–0.6)	−0.4	(−1.6–1.2)	−0.5	(−3.1–2.6)	0.27	0.20	0.48
Median catheter indwelling days (range)	2	(1–8)	1	(1–5)	1	(0–20)	0.06	0.03	0.50
Clot retention	4	(21)	0	(0)	7	(5)	0.11	0.03	>0.95
Surgical reintervention	2	(11)	0	(0)	3	(2)	0.49	0.11	>0.95
Transfusion	0	(0)	0	(0)	2	(1)	>0.95	>0.95	>0.95
Cardiovascular events	0	(0)	0	(0)	0	(0)	>0.95	>0.95	>0.95
Median days of hospital stay after surgery (range)	5	(3–12)	5	(3–7)	5	(2–14)	0.57	0.18	0.50
Re-admission	0	(0)	0	(0)	0	(0)	>0.95	>0.95	>0.95

*AP* antiplatelet agents, *AC* anticoagulant agents, *Hb* hemoglobin

**Table 5:** Cases with clot retention in AP/AC continuation group

	Sex	Medication	Tumor no.	Tumor size (cm)	pT stage	POD at catheter removal	Onset of clot retention (POD)	Treatment
Case 1	M	Aspirin	2	1.5	a	(−)	1	Surgical reinterventiom
Case 2	M	Aspirin	2	3	1	(−)	3	Surgical reinterventiom
Case 3	M	Aspirin	1	2	1	1	21	Bladder drainage
Case 4	M	Aspirin	6	2	1	2	3	Bladder drainage and irrigation

*AP* antiplatelet agents, *AC* anticoagulant agents, *no* number, *POD* postoperative day, *M* male

### Risk factors for clot retention in patients continuing AP/AC

We evaluated the risk factors for clot retention in patients continuing AP/AC drugs by comparing patients in the continuation group with (*n* = 4) and without clot retention (*n* = 15) (Table 6). Large tumors tended to be a risk factor for clot retention (*p* = 0.07), though the difference was not significant. However, age, tumor number, T classification at TURBT, and type and number of AP/AC agents were unrelated to clot retention (*p* > 0.1).

**Table 6:** Risk factors for clot retention in AP/AC continuation group

		Clot retention (+) *n* = 4, *n* (%)		Clot retention (−) *n* = 15, *n* (%)		*p* value
Median age (range)		77	(63–89)	77	(57–85)	0.76
Median tumor size, cm (range)		2	(1.5–3)	0.8	(0.5–3)	0.07
Tumor number	Single	1	(25)	10	(67)	0.26
	Multiple	3	(75)	5	(33)	
T classification at TURBT	pTa	1	(25)	7	(47)	0.43
	pT1	3	(75)	6	(40)	
	Min. pT2	0	(0)	2	(13)	
AP/AC drugs	Single AP	4	(100)	7	(47)	0.16
	Single AC	0	(0)	3	(20)	
	Combination	0	(0)	5	(33)	

*AP* antiplatelet agents, *AC* anticoagulant agents, *Min* minimum

## Discussion

The American Urologic Association and the International Consultation on Urological Disease produced a collaborative review of Anticoagulation and Antiplatelet Therapy in Urologic Practice [1], which stated that urologists need to understand the factors affecting the safe and effective use of AP and AC prophylaxis, as well as the risks posed by their withdrawal. These risks include venous and arterial thromboembolism, as well as major adverse cerebrovascular and cardiac events, which may be more life-altering than hemorrhage. Although the review also considered the management of AP/AC drugs during various urologic procedures, including shock wave lithotripsy, ureteroscopy with laser lithotripsy, percutaneous nephrolithotomy, laser prostatectomy, transurethral resection of the prostate, ultrasound-guided prostate biopsy, radical prostatectomy, and surgical renal procedures, it did not discuss the appropriate management of patients undergoing TURBT. A review of AP therapy in patients with coronary stents who underwent urologic surgery [8] included TURBT as a high risk procedure for bleeding. This review recommended that APs should be discontinued in patients at low thromboembolic risk undergoing TURBT, while elective surgery should be postponed if possible in patients at intermediate or high risk. In non-deferrable cases, aspirin should be continued if possible, P2Y12 inhibitors should be discontinued 5 days before surgery and resumed within 24–72 h with a loading dose, and bridge therapy with glycoprotein IIb/IIIa inhibitors is recommended if aspirin is discontinued, though these agents may be clinically unavailable, as in Japan. However, no replacement therapy has yet been validated prospectively [9].

International guidelines for percutaneous cardiovascular intervention advocate DAPT for ≥4 weeks after BMS implantation and for 6–12 months after DES implantation [2], and premature withdrawal of AP agents was shown to be related to a higher risk of cardiac ischemic or thromboembolic events associated with stent thrombosis [3]. This rare but life-threatening complication usually manifests as acute myocardial infarction, with a mortality of 10%–40%, though the incidence of stent thrombosis can be increased up to 90-fold following premature discontinuation of DAPT [10]. APs provide effective long-term secondary prevention of vascular events and ischemic stroke after acute or transient ischemic stroke. A systematic review of 287 randomized trials in patients at high risk of vascular occlusive events found that AP agents significantly decreased the risk of stroke by 31% [11]. Interruption of aspirin therapy was a significant risk factor for a stroke event within 4 weeks after aspirin discontinuation (odds ratio 3.4), with the main reasons for interrupting aspirin therapy being surgery, the treating physician’s decision that the therapy had no clear clinical benefit, and bleeding complications [12]. Five of 493 (1%) patients who stopped continuous ACs to allow the performance of dental procedures developed severe embolic complications, resulting in four deaths [4]. Overall, discontinuation of AP/AC agents is associated with severe thrombotic events and these drugs should be continued if at all possible.

Piccozi et al. recently demonstrated that continued use of aspirin monotherapy did not increase overall bleeding or reintervention risks in patients undergoing TURBT (2.8% in the aspirin group vs 1.9% in the control group) [5]. Camignani et al. assessed 12 patients receiving DAPT who underwent TURBT and demonstrated that no patients required reintervention for hemostatic purposes, but three (25%) of the 12 patients experienced clot retention after removal of the bladder catheter, all of which cases were resolved by replacing the catheter [7]. In the current study, we demonstrated that continuing AP/AC agents did not lead to increased blood loss or an increased incidence of blood transfusion compared with patients not taking or interrupting AP/AC agents. However, the incidence of clot retention was significantly increased in the AP/AC continuation group (21%) compared with the control group (5%). Interestingly, all four patients with clot retention received aspirin monotherapy, whereas patients taking AC agents or combined AP/AC agents did not experience clot retention. Large tumor size appeared to be a risk factor for clot retention, though the result was not statistically significant. Overall, these results suggest that TURBT can be performed in patients continuing to take AP/AC agents, including a combination of AP/AC agents, without increasing the risk of severe hemorrhage, though the risk of clot formation after surgery might be increased, especially in patients with large tumors. However, the incidence of complications in patients who interrupted AP/AC agents was similar to that in patients not taking these drugs, suggesting that it might be preferable to interrupt AP/AC agents in patients with a low thromboembolic risk, with a heparin bridge in patients taking ACs. However, the use of heparin-bridging therapy is controversial, and a recent study found that it did not reduce the risk of arterial thromboembolism compared with no bridging therapy in patients with stable nonvalvular atrial fibrillation [13]. In patients taking novel oral ACs, short-term interruption of these agents during the perioperative period might be possible because of their rapid offset and onset of anticoagulant activity [14].

There were several limitations to the present study. Notably, the retrospective nature of the study increased the risk of patient selection bias, and the sample size was small because of the small portion of patients taking AP and/or AC agents undergoing TURBT, which might underestimate the risk of taking these agents while undergoing TURBT. However, to the best of our knowledge, this is the first study to compare complications among patients who continued AP/AC, those who interrupted the treatment, and those not taking these agents, and to demonstrate that major bleeding complications were relatively rare, even in patients taking DAPT or a combination of AP and AC agents. However, further large prospective studies are therefore needed to verify these results.

## Conclusion

TURBT can be performed safely in patients who continue to use AP and/or AC agents without increasing the risks of severe hemorrhage and blood transfusion. However, the risk of clot retention after surgery may be increased in these patients.

## Abbreviations

AC: anticoagulant
AP: antiplatelet
BMS: bare metal stents
DAPT: dual antiplatelet therapy
DES: drug-eluting stents
M: male
Min: minimum
POD: postoperative day
TURBT: transurethral resection of bladder tumors
